# Efficacy of Biofeedback Therapy in Patients With Dyssynergic Defecation: A Hospital-Based Study in Eastern India

**DOI:** 10.7759/cureus.109606

**Published:** 2026-05-25

**Authors:** Habung Mobing, Pradeepta Sethy, Mihin Nania, Rahul Samanta, Prasanta Debnath

**Affiliations:** 1 Gastroenterology, Manipal Hospital, Kolkata, IND; 2 Anesthesia and Critical Care, Tomo Riba Institute of Health and Medical Sciences (TRIHMS), Naharlagun, IND

**Keywords:** biofeedback therapy, constipation, dyssynergic defecation, pelvic floor dyssynergia, treatment

## Abstract

Introduction: Dyssynergic defecation, a disorder of the uncoordinated defecation process, is a common cause of chronic constipation. Biofeedback is a self-regulation technique where, based on visual feedback, patients learn coordination of the pelvic floor muscles. This study aims to evaluate the effectiveness of biofeedback therapy in patients with dyssynergic defecation.

Methods: In a prospective observational study carried out over a period of one year from June 2023 to May 2024 in patients diagnosed with dyssynergic defecation, biofeedback therapy sessions were given for 30 minutes to one hour per session every two weeks for a maximum of six sessions over three months. Improvements in the Bristol stool form scale, constipation scoring system, and/or quality of life (The Medical Outcomes Study Short Form-36 or SF-36) were used to assess the response to therapy.

Results: Of the total 83 participants, 49 (59.04%) were male, and the median age was 43 years. Type I dyssynergia was the most common type. Thirty (36.1%), 37 (44.6%), and nine (10.8%) participants had improvement in Bristol stool form, constipation severity score, and SF-36 score, respectively. Bristol type 3 was the most common stool form at the first visit, while type 4 was the predominant stool form at the last visit. An improvement in Bristol stool form was seen in 30 (36.1%) participants. The average constipation scores at the first and last visits were 15.7±1.86 and 13.4±2.79, respectively (P<0.05), with improvement seen in 37 (44.6%) participants. The average SF-36 scores at the initiation of therapy and the last visit were 47.7±4.15 and 48.08±3.84, respectively (P>0.05), with improvement seen in only nine (10.8%) participants. Post-biofeedback therapy, an improvement in either of the three parameters, i.e., Bristol stool form, constipation severity score, or SF-36 score, was seen in 59 (71.1%) participants, of whom 37 (62.71%) responded to the therapy with two sessions.

Conclusion: Biofeedback therapy is beneficial in patients with dyssynergic defecation, with significant improvements in stool form and constipation-related symptoms.

## Introduction

Constipation is a common gastrointestinal disorder encountered in daily clinics with an estimated global prevalence of 12%-19% [1]. It is also a common problem in Indian communities and in clinical practice [2]. Constipation is labeled as acute if symptom duration is less than one week, while for chronic constipation, symptoms should last for more than three months [3].

Constipation may be primary or secondary; slow-transit or normal-transit. Incoordination between the pelvic floor muscles and anal sphincter muscles may cause a form of functional obstruction and hinder the process of defecation [4]. Though there are many terms used to describe this disorder, the term dyssynergic defecation has been adopted by the Rome Criteria, as the main pathophysiology involves incoordination in the defecation process [5].

Constipation is associated with increased psychological distress. Dyssynergia may follow events like pregnancy, trauma, or back injury. No discernible cause can be established in the majority of patients [6].

Symptoms may include excessive straining during defecation, a sensation of incomplete evacuation after defecation, a sense of fullness in the abdomen, and the use of digital maneuvers to evacuate stool [7]. These patients may also report two or fewer stools per week, laxative dependence, relief of abdominal pain after defecation, or alternating diarrhea and constipation [8]. Biofeedback therapy has also been shown to be an effective treatment for those patients having overlapping IBS features, levator ani syndrome, and solitary rectal ulcer syndrome [5,9,10]. Apart from abdominal and defecation-related symptoms, these patients also have associated psychiatric disorders and a poor quality of life [11].

The presence of any concomitant metabolic or pathologic disorder should be ruled out before labeling a diagnosis of dyssynergic defecation, which can be done by obtaining a detailed clinical history, physical examination, including perianal and digital rectal examination, and flexible sigmoidoscopy. Commonly used diagnostic modalities to detect dyssynergia are anorectal manometry, balloon expulsion test, defecography and magnetic resonance defecography, and colonic transit study [5].

Anorectal manometry is used for diagnosing dyssynergic defecation, along with providing additional information on its subtypes. It is also helpful in assessing rectal sensation, reflexes, and compliance [12]. Diagnosis is based on findings of abnormal manometry and rectal balloon expulsion testing [13]. Based on manometry findings, four types of dyssynergic defecation have been recognized, which include Type I (rise in intra-abdominal pressure along with a paradoxical increase in anal sphincter pressure); Type II (no increase in intra-rectal pressure but exhibiting a paradoxical anal sphincter contraction); Type III (increase in intra-rectal pressure but no decrease in anal sphincter pressure); and Type IV (no increase in intra-rectal pressure along with absent or incomplete anal sphincter relaxation) [5].

The management of dyssynergic defecation includes lifestyle changes, drugs, and biofeedback therapy. Refractory cases may require invasive therapies, such as botulinum toxin injection and surgery. General measures include avoiding drugs that cause constipation, adequate fluid and fiber consumption, physical activity, timed toilet training, and use of laxatives [14].

Biofeedback is a form of guided self-regulation technique where patients teach themselves to control body processes using auditory and visual feedback from a specialized device in the presence of a trained biofeedback therapist. Various conditions are being effectively treated with biofeedback therapy [15].

Patients should be informed about anorectal dysfunction and biofeedback therapy before initiating treatment. The training emphasizes improving pushing effort and the use of abdominal and anorectal muscles based on visual feedback and guidance of the therapist. About four to six sessions of 30-60 minutes each are given to the patient, although there is no standard number or duration for these sessions [5].

Despite the high cost of this therapy, studies have shown sustained improvement in patient symptoms for up to two years. For these reasons, biofeedback training has become the preferred treatment for functional defecation disorder in recent years [16].

Data from western India showed that dyssynergic defecation was present in 40% of patients with chronic constipation [17]. Despite a high prevalence, there is an unavailability of established guidelines to manage dyssynergic defecation in our country, which may be attributed to a lack of information about biofeedback therapy among patients. Also, this therapy is time-consuming, mandates proper communication with the patient, and requires multiple visits to the hospital, thus limiting its acceptability among the Indian population [18].

There are very few studies related to biofeedback therapy and its implications for dyssynergic defecation in the Indian population. This study may help analyze the efficacy of biofeedback therapy on dyssynergic defecation, especially in the eastern part of India and the country as a whole. It will also contribute immensely to increasing awareness and improving its acceptability in the Indian setting.

This article was previously presented as an oral paper at the 65th Annual Conference of the Indian Society of Gastroenterology, ISGCON 2024, on December 7, 2024.

## Materials and methods

Study setting

This study followed a prospective observational design and was carried out over a period of one year, extending from June 2023 through May 2024, in patients aged 18 years or older diagnosed with dyssynergic defecation using anorectal manometry. Those not willing to participate in the study, patients with secondary causes of constipation, uncooperative patients, and pregnant women were excluded from the study. Secondary causes of constipation were ruled out using routine blood investigations, flexible sigmoidoscopy, colonoscopy, and imaging techniques (CT or MRI). The study was carried out after obtaining informed written consent from the participants and clearance from the Scientific Research Committee (SRC/Gastro-011/2023) and the Ethics Committee (CREC/2023/JUL/1(iv)).

Biofeedback therapy

Biofeedback training sessions were given by a therapist using the Royal Melbourne Hospital High Resolution Manometry & 16 Channel Water Perfusion System. The subject was positioned in the left lateral decubitus position with the manometry probe in situ and was instructed to take a deep diaphragmatic breath and bear down as though he was passing stool while viewing the monitor or listening to instructions from the therapist. The therapist also supervised the breathing techniques and postural alignment of the patient. After 10 such maneuvers, 60 mL of air was used to distend the balloon so that the patient could get the sense of fullness in the rectum or an urge to pass stool. This was followed by encouraging the patient to push or bear down and try to pass the balloon out. The maneuvers were repeated five times. Along with rectal coordination training, training on simulated defecation was provided by filling the balloon placed in the rectum with 50 mL of water. The patient was guided on how to relax the pelvic floor muscles in a correct posture and use proper breathing technique so that the patient was able to expel the balloon. Each session lasted for 30 minutes to one hour. These sessions were provided every two weeks for a maximum of six therapy sessions over three months.

Bristol stool form scale

For assessment of constipation, the Bristol stool form scale was used, which described seven stool forms based on stool form and consistency: type 1 (separate hard lumps like nuts); type 2 (sausage-shaped, but lumpy); type 3 (like a sausage but with cracks on its surface); type 4 (like a sausage or snake, smooth and soft); type 5 (soft blobs with clear-cut edges); type 6 (fluffy pieces with ragged edges, a mushy stool); and type 7 (watery, no solid pieces, entirely liquid) [19].

Constipation scoring system

Based on a study conducted by Agachan F et al., we calculated a score on the basis of a constipation symptom-related questionnaire, which varied from a minimum score of 0, suggestive of a normal value, to a maximum score of 30 signifying severe constipation [20].

Quality of life assessment

Developed by Ware JE et al., the Medical Outcomes Study Short Form-36 (SF-36) was used to measure multidimensional quality of life using a questionnaire assessing eight domains, including physical functioning, role-physical, bodily pain, general health, vitality, social functioning, role-emotional, and mental health, such that a higher score signified better quality of life [21].

Bristol stool form scale, constipation scoring system, and SF-36 were assessed at the beginning of therapy and subsequent follow-up. A patient was followed up until an improvement was noted in either one of the parameters or he/she had completed a maximum of six therapy sessions. Total confidentiality was maintained by coding the patient’s data.

A sample size of 88 was arrived at using the formula 4PQ/L2, where P is prevalence=6%; Q=100-P=94; and L is the absolute allowable error=5% at 95% confidence interval.

Statistical analysis

Depending on distribution, data were expressed as mean±SD and as percentages. Kolmogorov-Smirnov analysis was used to check if the data followed a normal distribution. The Fisher's exact test or the chi-square test was used to check the significance of differences between frequency distributions of data. Friedman’s test and the Wilcoxon matched-pairs test were used for correlation analysis. P-value <0.05 was considered statistically significant. The statistical software SPSS version 26 (IBM Corp., Armonk, NY) was used for the analysis.

## Results

**Figure 1:**
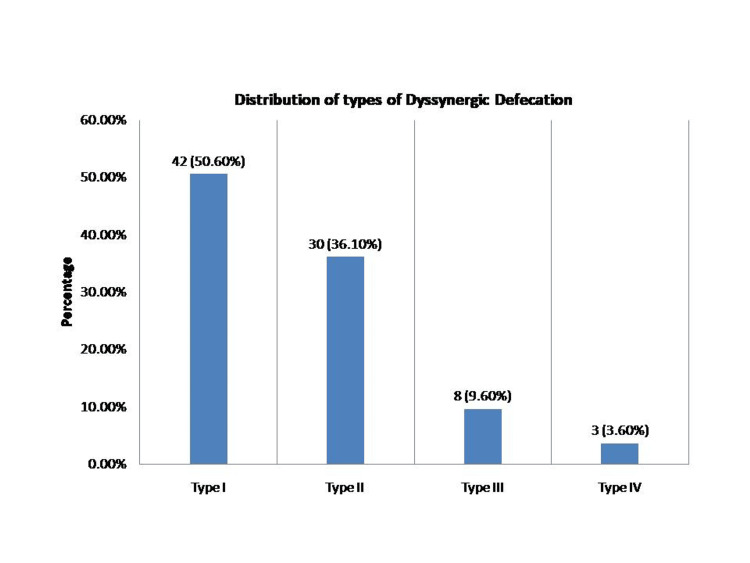
Distribution of types of dyssynergic defecation (n=83) The data has been represented as n (%).

A total of 88 patients with dyssynergic defecation fulfilled the inclusion criteria and were enrolled in the study. However, five of them were lost to follow-up, i.e., failed to complete a maximum of six therapy sessions despite no improvement in any of the parameters (Bristol stool form, constipation severity score, or SF-36 score). Of the remaining 83 participants, 49 (59.04%) were male, and the median age was 43 years. Type I dyssynergia was the most common type seen in 42 (50.6%) participants, followed by Type II, Type III, and Type IV, i.e., 30 (36.1%), eight (9.6%), and three (3.6%), respectively (Figure 1).

At the first visit, the most common Bristol stool form was type 3 (38.6%), while at the last visit, type 4 (37.3%) was the predominant type. This finding was statistically significant using Friedman’s test (P=0.001). Only 30 (36.1%) participants had improvement in Bristol stool form (Table 1, Table 2).

**Table 1:** Distribution of Bristol stool form types at the first and last visits (n=83) The data has been represented as n. P-value was determined using Friedman’s test, and a value <0.05 was considered statistically significant.

Bristol stool form types	Number of patients with Bristol stool form at 1st visit (n=83)	Number of patients with Bristol stool form at last visit (n=83)	P-value
Type 1	4	0	0.001
Type 2	14	4	
Type 3	32	27	
Type 4	18	31	
Type 5	10	16	
Type 6	4	4	
Type 7	1	1	

**Table 2:** Frequency of improvement in Bristol stool form (n=83) The data have been represented as n (%).

Frequency of improvement in Bristol stool form	Number of patients (%)
Improvement	30 (36.10%)
No improvement	53 (63.90%)

On evaluation of the constipation severity score, the average constipation score at the first visit was 15.7±1.86, while at the last visit, the average score was 13.4±2.79. This finding was statistically significant (P<0.05) using the Wilcoxon matched-pairs test. Thirty-seven (44.6%) participants had improvement in the constipation severity score (Table 3).

**Table 3:** Frequency of improvement in the constipation severity score (n=83) The data have been represented as n (%).

Frequency of improvement in the constipation severity score	Number of patients (%)
Yes	37 (44.60%)
No	46 (55.40%)

On assessment of quality of life by SF-36, the average score at the initiation of therapy was found to be 47.7±4.15, while at the last visit, the average score was 48.08±3.84. However, this finding was not statistically significant (P>0.05) in the Wilcoxon matched-pairs test. An improvement in the SF-36 score was seen in only nine (10.8%) participants (Table 4).

**Table 4:** Frequency of improvement in the SF-36 score (n=83) The data have been represented as n (%) SF-36, The Medical Outcomes Study Short Form-36

Frequency of improvement in the SF-36 score	Number of patients (%)
Improvement	9 (10.8%)
No improvement	74 (89.2%)

However, an improvement in either of the three parameters, i.e., Bristol stool form, constipation severity score, or SF-36 score, was seen in 59 (71.1%) participants (Table 5).

**Table 5:** Frequency of improvement in any one of the parameters (Bristol stool form scale, constipation severity score, or SF-36 score) (n=83) The data have been represented as n (%). SF-36, The Medical Outcomes Study Short Form-36

Improvement in any one of the parameters (Bristol stool scale, constipation severity score, or SF-36)	Number of patients (%)
Improvement	59 (71.10%)
No improvement	24 (28.90%)

Among the 59 participants who had an improvement in either one of the parameters, 37 (62.71%) responded to the therapy with two sessions of biofeedback therapy, while three (5.08%) responded after a maximum of six sessions. The highest proportion of participants with improvement in any one of the parameters was seen in patients with Type III dyssynergia, where six out of eight participants (75%) experienced improvement, followed by Type I dyssynergia, where 31 (73.8%) out of 42 participants experienced improvement. Type II and Type IV had similar proportions of participants (66.7%) who experienced improvement, i.e., 20 out of 30 and two out of three, respectively. However, these outcomes were not statistically significant (P>0.05) in the chi-square test.

## Discussion

In this study, the majority of the participants were male, and the median age was 43 years. A study done in the Indian population conducted by Jain M et al. in 2018 reported that the median age among patients with dyssynergic defecation was 45.6 years, and the majority, i.e., 78 (73.6%) out of 106 patients, were males, which were similar to the findings in this study [22].

Type I dyssynergia was the most common type of dyssynergia seen in this study, which was followed by Type II dyssynergia, while the least common type was Type IV dyssynergic defecation. Zhao Y et al. in 2019 showed in their study that Type I was the most common type of dyssynergia, followed by Type II dyssynergia, while Type III dyssynergia was the least common type [23].

This study showed that type 3 Bristol stool form was the most common type of Bristol stool form during the first visit, while at the last visit, type 4 was seen in the majority of the participants. This improvement in Bristol stool form was statistically significant but was seen in only 30 (36.1%) participants. Similar to this study, a prospective study conducted by Özkütük N et al. in 2021 reported that before therapy, most patients had type 3 stool form, and stool form improved to type 4 in 41.6% of patients in the group who received biofeedback therapy in comparison to the control group [24].

The average constipation severity score prior to the initiation of biofeedback therapy was 15.7±1.86, while at the last visit, the average score was 13.4±2.79. The improvement in constipation severity score was statistically significant and was seen in 37 (44.6%) participants. This constipation severity score was also used in another study conducted by Sagae UE et al. in 2012 to evaluate the efficacy of biofeedback therapy in patients with chronic constipation, which showed a significant improvement in post-biofeedback therapy constipation scores compared with pre-biofeedback scores [25].

An improvement in the SF-36 score was seen in only nine (10.8%) participants. The average SF-36 score at the initiation of therapy was 47.7±4.15, while at the last visit, the average score was 48.08±3.84. However, this change in score was not statistically significant. Şahin M et al. in 2015 in their study showed a significant improvement in post-therapy SF-36 scores when compared with pre-therapy values [26].

An improvement in either of the parameters, i.e., Bristol stool form, constipation severity score, or SF-36 score, after receiving biofeedback therapy was seen in 59 (71.1%) participants. A study conducted by Verma A et al. in 2017 to evaluate the effect of biofeedback therapy on patients with fecal evacuation disorder showed that 62% of patients had satisfactory symptomatic improvement during a one-month follow-up period [27]. Another study conducted in the Indian population by Agarwal A et al. in 2024 reported that a significant improvement in overall symptoms was noted in 74.6% of subjects post-biofeedback therapy [28].

Though the recommended number of biofeedback therapy sessions is about four to six sessions [29], the results of this study showed that among the responders, 62.71% of participants responded to the therapy with two sessions of biofeedback therapy, while 5.08% of participants required a maximum of six sessions for a positive outcome. This discrepancy in the lower number of sessions required for response to therapy in this study might be due to the shorter duration of the study period. Also, patients were not followed up once they experienced improvement post-therapy, irrespective of the number of sessions.

In a study conducted by Abdinoor A et al. in 2015, patients with Type I dyssynergic defecation had a better response to biofeedback therapy compared to other subtypes, while another study by Agarwal A et al. in 2024 found that the efficacy of biofeedback therapy was equal, irrespective of the type of dyssynergic defecation [28,30]. However, in this study, patients with Type III dyssynergia had the highest proportion of participants with overall improvement. However, this finding was not statistically significant.

The limitation of this study was that it was conducted in a single center and in a small study population, with 5.7% of patients lost to follow-up. Since there was a lack of a control group for comparison of outcomes, the possibility of a placebo effect or observer bias cannot be ruled out. Effects of confounders associated with chronic constipation, such as lifestyle, diet, medications, and other comorbidities, were not taken into account. Although the assessment of the efficacy of therapy was based on scales or scoring systems, its effect on individual clinical symptoms and manometric findings was not studied. Finally, the study period was short, and the long-term effects of the therapy were not analyzed. Hence, a larger multicenter randomized controlled study will be required to substantiate the findings of this study. Nevertheless, the outcome of this study shows that a significant number of participants with dyssynergia undergoing biofeedback therapy had improvement, especially in stool form and constipation-related symptoms. In conclusion, this therapy can be recommended as part of the treatment protocol for dyssynergic defecation in the Indian population.

## Conclusions

Dyssynergic defecation is prevalent in patients suffering from chronic constipation. Though biofeedback therapy is an established treatment modality for dyssynergic defecation, there are limited studies related to biofeedback therapy and its impact on dyssynergic defecation in the Indian population. Also, there is restricted acceptability for this therapy among Indian patients, probably due to its unavailability, affordability, lack of motivation, poor compliance, and discomfort (invasiveness). The beneficial effects of biofeedback therapy on dyssynergic defecation in this prospective observational study were noted in 71.1% of patients, with significant improvements observed in Bristol stool form and constipation severity score. Findings of this study emphasize incorporating biofeedback therapy as part of the dyssynergic defecation treatment protocol in the Indian population. However, further multicenter randomized controlled trials with sham biofeedback or standard care control arms are needed.
